# From Mouth to Model: Combining *in vivo* and *in vitro* Oral Biofilm Growth

**DOI:** 10.3389/fmicb.2016.01448

**Published:** 2016-09-21

**Authors:** Barbara Klug, Elisabeth Santigli, Christian Westendorf, Stefan Tangl, Gernot Wimmer, Martin Grube

**Affiliations:** ^1^Institute of Plant Sciences, University of GrazGraz, Austria; ^2^Department of Dental Medicine and Oral Health, Division of Oral Surgery and Orthodontics, Medical University of GrazGraz, Austria; ^3^Karl Donath Laboratory for Hard Tissue and Biomaterial Research, Department of Oral Surgery, Medical University of ViennaVienna, Austria; ^4^Austrian Cluster for Tissue RegenerationVienna, Austria; ^5^Department of Dental Medicine and Oral Health, Division of Preventive and Operative Dentistry, Periodontology, Prosthodontics and Restorative Dentistry, Medical University of GrazGraz, Austria

**Keywords:** native oral biofilm, *in vitro* growth, dental splint, human enamel-dentin slabs, live/dead staining, 454 pyrosequencing

## Abstract

**Background:** Oral biofilm studies based on simplified experimental setups are difficult to interpret. Models are limited mostly by the number of bacterial species observed and the insufficiency of artificial media. Few studies have attempted to overcome these limitations and to cultivate native oral biofilm.

**Aims:** This study aimed to grow oral biofilm *in vivo* before transfer to a biofilm reactor for *ex situ* incubation. The *in vitro* survival of this oral biofilm and the changes in bacterial composition over time were observed.

**Methods:** Six human enamel-dentin slabs embedded buccally in dental splints were used as biofilm carriers. Fitted individually to the upper jaw of 25 non-smoking male volunteers, the splints were worn continuously for 48 h. During this time, tooth-brushing and alcohol-consumption were not permitted. The biofilm was then transferred on slabs into a biofilm reactor and incubated there for 48 h while being nourished in BHI medium. Live/dead staining and confocal laser scanning microscopy were used to observe bacterial survival over four points in time: directly after removal (T0) and after 1 (T1), 24 (T2), and 48 h (T3) of incubation. Bacterial diversity at T0 and T3 was compared with 454-pyrosequencing. Fluorescence *in situ* hybridization (FISH) was performed to show specific taxa. Survival curves were calculated with a specially designed MATLAB script. Acacia and QIIME 1.9.1 were used to process pyrosequencing data. SPSS 21.0 and R 3.3.1 were used for statistical analysis.

**Results:** After initial fluctuations at T1, survival curves mostly showed approximation of the bacterial numbers to the initial level at T3. Pyrosequencing analysis resulted in 117 OTUs common to all samples. The genera *Streptococcus* and *Veillonella* (both *Firmicutes*) dominated at T0 and T3. They make up two thirds of the biofilm. Genera with lower relative abundance had grown significantly at T3. FISH analysis confirmed the pyrosequencing results, i.e., the predominant staining of *Firmicutes*.

**Conclusion:** We demonstrate the *in vitro* survival of native primary oral biofilm in its natural complexity over 48 h. Our results offer a baseline for cultivation studies of native oral biofilms in (phyto-) pharmacological and dental materials research. Further investigations and validation of culturing conditions could also facilitate the study of biofilm-induced diseases.

## Introduction

At the beginning of the twenty-first century, natural biofilm modeling still poses a great challenge. The biofilm lifestyle of oral bacteria is difficult to simulate, as a normal human oral microbiome comprises more than 700 different bacterial taxa (Aas et al., 2005). The composition of the bacterial community and its spatial distribution have been studied in various ways to reveal a highly structured organization of the biofilm. The matrix surrounding and protecting the biofilm has been compared to a bacterial “house” (Flemming et al., 2007) that provides structures like channels for nutrition supply and communication. Varying surrounding conditions inside a biofilm modify bacterial lifestyles and lead to adaptations, e.g., they render bacteria more resistant to antibiotics (Høiby et al., 2011). Under healthy conditions, this oral ecosystem is in a homeostatic state (Marsh, 2006), but this does not imply uniformity across the microbiome composition. Quite the opposite is the case, as each human being hosts a genuine oral microbiome with an individual bacterial composition on the whole and within each oral compartment (Arweiler et al., 2004; Trajanoski et al., 2013; Langfeldt et al., 2014), e.g., the salivary microbiome composition differs from that of the subgingiva, and the tongue's microbiome is different from that of the cheek (Aas et al., 2005; Simón-Soro et al., 2013). Moreover, our knowledge about natural co-occurrence patterns and patterns of mutual exclusion is still incomplete (Human Microbiome Project Consortium, 2012; Segata et al., 2012). In addition to the bacterial composition, factors outside the biofilm can have an influence on it. They include parameters such as shear stress through salivary flow, natural temperature oscillations, pH value changes, host immunity factors, stress or dietary variation, all of which still need to be fully explored and understood (Rittman, 1982; van Houte et al., 1982; Saunders and Greenman, 2000; Picioreanu et al., 2001; Wimmer et al., 2005; Al-Ahmad et al., 2007; De Filippo et al., 2010; Fierer et al., 2010; Hajishengallis, 2010; Schlafer et al., 2011). It is almost impossible to include all these factors as model parameters, especially considering the fact that they have not yet all been identified. As a result, most of the experimental setups in *in vitro* models so far have generally focused on a reduced number of these parameters. However, the processes and interactions in complex oral biofilms are difficult to interpret based on simplified experimental setups. Many bottom up assays in microbiology are still limited by two main factors: firstly, the number of bacterial species included and, secondly, the artificial media used to feed the biofilm. They explore individual functional roles and inter-individual interactions (Hansen et al., 2000; Mazumdar et al., 2008; Periasamy and Kolenbrander, 2009; Standar et al., 2010; Agostinho et al., 2011). Alternatively, a top down approach compiles information about structure, spatial distribution and community composition (Filoche et al., 2010; Zijnge et al., 2010; Klug et al., 2011; Edlund et al., 2013; Nyvad et al., 2013; Jorth et al., 2014; Sintim and Gürsoy, 2015; Zheng et al., 2015). Also, advanced experimental setups that include different media and several bacterial species only insufficiently reflect natural conditions, particularly those in the oral habitat. Combining all these setups can lead to better models that realistically mimic natural conditions. Even if the biological parameters cannot be fully reconstructed, tracking the bacterial composition of multispecies biofilms can provide new insights into their interactions. One important step is to enable the transfer of native oral biofilm to experimental setups, and to keep it vital and diverse under laboratory conditions. In the present study, we introduce a standardized workflow to grow native oral biofilm *in vivo*, to transfer this biofilm into an *in vitro* environment and to keep it alive in that environment. With this “mouth to model” procedure, we demonstrate the survival of primary oral biofilm grown natively under simplified laboratory conditions and the changes that take place in the bacterial composition.

## Materials and methods

### *In vivo* oral biofilm growth—study participants and dental splints

This study was approved by the institutional review board at the Medical University of Graz. Written informed consent was obtained from all study participants in accordance with the Declaration of Helsinki. The study design and the role of study participants were communicated to them in advance.

**Figure 1:**
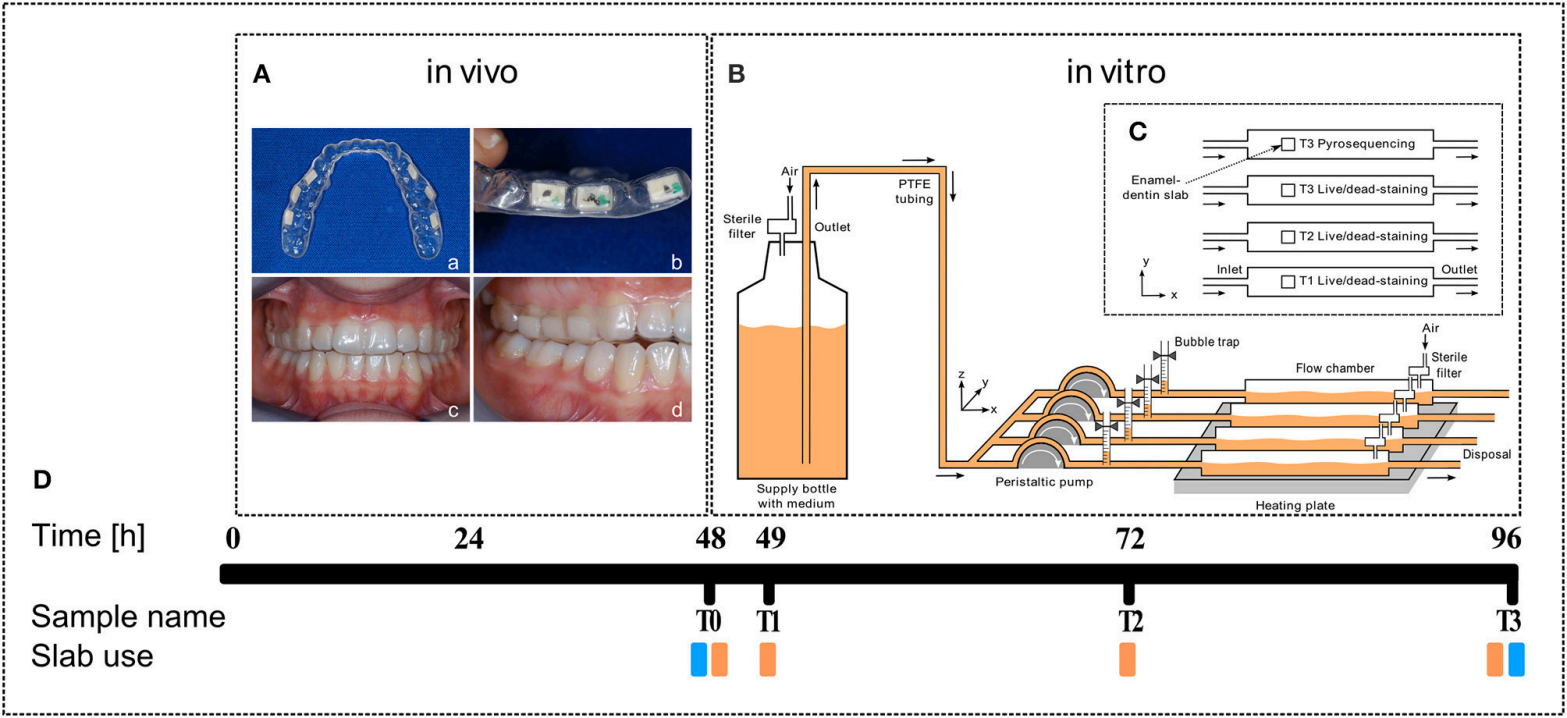
**Experimental outline. (A)** Dental splint with six human enamel-dentin slabs (white squares, **a**, top view; **b**, side view) fixed in the upper jaw (**c**, front view; **d**, side view). **(B)** Sketch of the biofilm reactor setup (from left to right): Supply bottle filled with BHI medium (orange), peristaltic pump, bubble trap, DFR 110 biofilm reactor on heating plate (34°C), arrows indicate medium flow direction. **(C)** Top view sketch of the enamel-dentin slab distribution in biofilm reactor chambers. **(D)** Timeline and slab use (orange, live/dead staining; blue, pyrosequencing).

Twenty-five dental students aged between 20 and 25 years were recruited via notice-board at the School of Dentistry of the Medical University of Graz. Prior to enrollment a short history was taken to ensure that the following inclusion criteria were met: non-smoker, good general health, no present medication and no antibiotic intake 3 months prior to this study. Due to possible hormonal shifts, only male students were included. For each study participant a dental splint was fitted individually to the upper jaw including six standardized (6 × 4 mm) enamel-dentin slabs for native oral biofilm collection. The slabs had been prepared from patients' teeth that had been extracted for medical reasons at the outpatient clinic of the University Department of Dentistry in Graz. The dentin-enamel slabs were sterilized and then cut, grinded and polished at the Karl Donath Laboratory for Hard Tissue and Biomaterial Research in Vienna. After that, they were integrated buccally in individually fitted dental splints facing the surface of the teeth (Figures 1Aa,b), leaving a small gap between slab and tooth (Figures 1Ac,d). Participants were asked to wear the dental splint continuously for 48 h. They were not allowed to drink alcohol or brush their teeth during this time to guarantee undisturbed biofilm growth.

### *In vitro* biofilm growth–biofilm reactor

Dental splints were removed carefully directly in the lab after 48 h and immediately placed into a pre-warmed Brain Heart Infusion (BHI, Roth, Austria) medium. Permanently covered with BHI, the enamel-dentin slabs were consecutively clipped out and transferred into the DFR 110 biofilm reactor (Biosurface Technologies Corporation, Montana, USA). Biofilm was then incubated for another 48 h at 34°C with a BHI flow rate of 0.2 ml/min (Figures 1B,C). Measurements were performed at four points in time: T0—directly after removal from the mouth, T1—after 1 h incubation, T2—after 24 h incubation, and T3—after 48 h incubation (Figure 1D).

### Live/dead staining

Biofilm on enamel-dentin slabs was stained with LIVE/DEAD® BacLight™ Bacterial Viability Kit (Molecular Probes®) according to protocol. Slabs were fully submerged in the staining solution [Syto 9, green fluorescence, and Propidium iodide (PI), red fluorescence] for 20 min at room temperature. After washing with sterile ddH_2_O, the biofilm was analyzed directly on the slabs with a Leica TCS-SP confocal laser scanning microscope (CLSM). The slabs were covered with water, and water-immersible objectives (HCX APO L 20x/0.5 W UVI/D 3.5 and HCX APO L 63x/0.90 W) were used to generate stack data. Filters were set at 501–531 nm for Syto 9 and 600–672 nm for PI. At least five stacks at random positions were recorded for each slab.

### Image processing

The set of all non-zero pixels of an individual confocal stack was clustered into four different clusters, using the kmeans function in MATLAB® (Statistics and Machine Learning Toolbox). The cluster with the lowest mean represented the background and noise, and was subtracted from the image stack, whereas the three remaining clusters represented the foreground. Occasionally present yeast and oral mucosa cells were manually selected and removed from the binary masks of both corresponding confocal stacks. The area covered by stained bacteria was simply calculated as the count of all non-zero pixels in the entire binarized confocal stack. As a mixed environmental biofilm, a fraction of cells was labeled by both dyes contained in the LIVE/DEAD® BacLight™ Bacterial Viability Kit. Double-labeled pixels (orange) were always counted as dead and removed from the corresponding binary mask of living bacteria. Finally, for each corresponding pair of confocal stacks, the fraction of living and dead bacteria was computed, with 100% being the sum of both.

### DNA extraction for microbial community analysis

Enamel-dentin slabs (T0 and T3) were glued into the lids of 1.5 ml Eppendorf tubes with epoxy resin adhesive that covered all sides except the standardized surface with the biofilm on it. Sterile and DNA-free glass beads and 200 μl ultra-pure water were inserted, and the biofilm in the vials was shredded for 2 min. For total DNA isolation, the lysate was mixed with 380 μl of MagNA Pure Bacteria Lysis Buffer (Roche Applied Science, Mannheim, Germany) together with 20 μl of proteinase K solution (20 mg/ml) and incubated at 65°C for 10 min. Proteinase K was heat-inactivated at 95°C for another 10 min. The liquid samples were transferred to MagNA Pure Compact Sample Tubes. DNA isolation was performed on the MagNA Pure Compact instrument according to manufacturer instructions using the MagNA Pure Compact Nucleic Acid Isolation Kit I and following the bacteria purification protocol (Roche Diagnostics, Mannheim, Germany). The DNA was eluted in 50 μl elution buffer and stored at −20°C pending further processing.

### 454-pyrosequencing and data analysis

A 505 bp fragment targeting the V1-V3 region of the 16S rRNA gene was amplified using FLX 454 one way read fusion primers F27—AGA GTT TGA TCC TGG CTC AG and R534—ATT ACC GCG GCT GCT GGC (Watanabe et al., 2001; Baker et al., 2003). QPCR was used to ensure equal DNA amounts for the FLX 454 run. All samples were run on the same plate to exclude bias. Purified amplicon DNAs were quantified using the Quant-iT PicoGreen kit (Invitrogen, Carlsbad, CA) and pooled for pyrosequencing.

Roche GS FLX raw sequences were denoised and quality-checked using Acacia (Bragg et al., 2012). A minimum length of 150 bases was used with a Phred score of more than 25. No ambiguous bases and two-bases maximum edit distance in the forward primer were allowed. Acacia also assigned sequences to the according tag and trimmed the primer and barcode sequences. The Quantitative Insights Into Microbial Ecology (QIIME) pipeline version 1.9.1 was then used for downstream analysis (Caporaso et al., 2010b). Sequences were clustered into Operational Taxonomic Units (OTUs) with a 97% identity. Alignment of representative sequences was then performed with Greengenes 16S rRNA gene database using pyNAST (Caporaso et al., 2010a). FastTree was used to generate phylogenetic trees (Price et al., 2009). Taxonomies were assigned with the RDP Classifier (Wang et al., 2007). ChimeraSlayer implementation was used to perform chimera check on aligned representative sequences. Alpha-diversity estimates were then calculated using PD whole tree (Chao et al., 2010), Shannon and chao1 (Chao, 1984) metrics. Finally, beta-diversity was evaluated using Principal Coordinate Analysis (PCoA) plots based on unweighted UniFrac distance matrices (Lozupone et al., 2011). Rarefaction to the read size with the lowest number was performed to adjust samples for UniFrac analysis.

All statistical analyses were performed using SPSS version 21.0 (SPSS Inc., Chicago, IL). Shapiro Wilk's Test was used to test for normal distribution of the data. Data were presented as median, and 25th and 75th percentile. Wilcoxon signed-rank tests with Bonferroni correction for multiple comparisons were used for comparing T0 and T3. All reported values of *p* < 0.05 were considered statistically significant after Bonferroni correction.

Heat maps of the relative abundances were created in R version 3.3.0 using the phyloseq package and the plot_heatmap function within (Rajaram and Oono, 2010; McMurdie and Holmes, 2013). Correspondence analysis was performed in R 3.3.1 using the Vegan Package 2.4. OTUs with zero counts at one of the points in time were removed to ensure that previously reported bias does not occur in the plots (Zuur et al., 2007). Site-specific scaling was chosen for the biplots. Log_2_ fold change was calculated with Matlab R2016 on OTU level plotting the respective genera subsequently. Absolute abundances from the heat map data were used and 7 OTUs excluded as their average abundance in one of the points in time was zero.

### Fluorescence *In situ* hybridization

For fixation of the biofilm, enamel-dentin slabs from T0–T3 were inserted into ice-cold 4% PFA solution directly after removal. Slabs were then incubated for 8 h at 4°C. Subsequently, PFA was removed and slabs were washed two to three times with 1 × PBS. Samples were stored in 1 × PBS/96% ethanol (v/v), unless they had been used immediately. After that, Fluorescence *In Situ* Hybridization (FISH) was performed in 1.5 ml vials as described previously (Klug et al., 2011). Probes Bac303 (staining most *Bacteroidaceae* and *Prevotellaceae*, and some *Porphyromonadaceae*), EUB338mix (EUB338, EUB338II, EUB338III staining most bacteria), and LGC354mix (LGC354A, -B and -C staining *Firmicutes*) were used (Loy et al., 2007). FISH analysis was performed on the TCS-SP CLSM, as was the live/dead analysis. Filters were set at 500–535 nm for FITC, 560–612 nm for Cy3, and 656–721 nm for Cy5. AMIRA 3D software (FEI, Europe) was used to generate 3D reconstructions of the confocal stack data.

## Results

### Survival of bacteria

**Figure 2:**
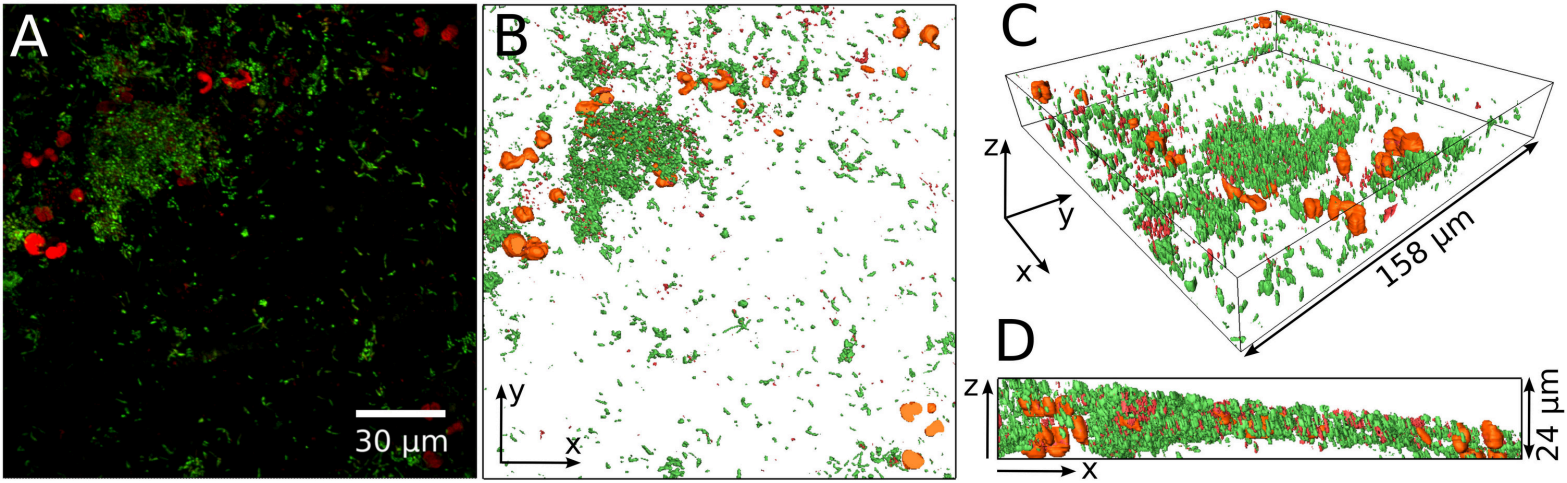
**Example of yeast cells embedded in the bacterial biofilm**. Maximum projection of the entire confocal stack of a life/dead stained biofilm **(A)**. Green, living bacteria; red, dead bacteria and yeast. The prominent red cells are probably yeast cells and are visualized in orange in a 3D reconstruction of the biofilm **(B–D)**. **(B)** Gives the top view while **(C,D)** show side views of the 3D reconstruction.

An example of CLSM data from a biofilm containing yeast is given in Figure 2. A maximum projection of a CLSM stack is shown in Figure 2A with living cells in green, and dead and yeast cells both in red. The 3D reconstruction of the same stack is presented in Figures 2B–D. The large orange structures in the 3D reconstruction are presumably yeast cells and thus they were excluded from the analysis. Supplementary Video 1 shows the performance of our MATLAB script cleaning the data for evaluation of the live/dead ratio.

**Figure 3:**
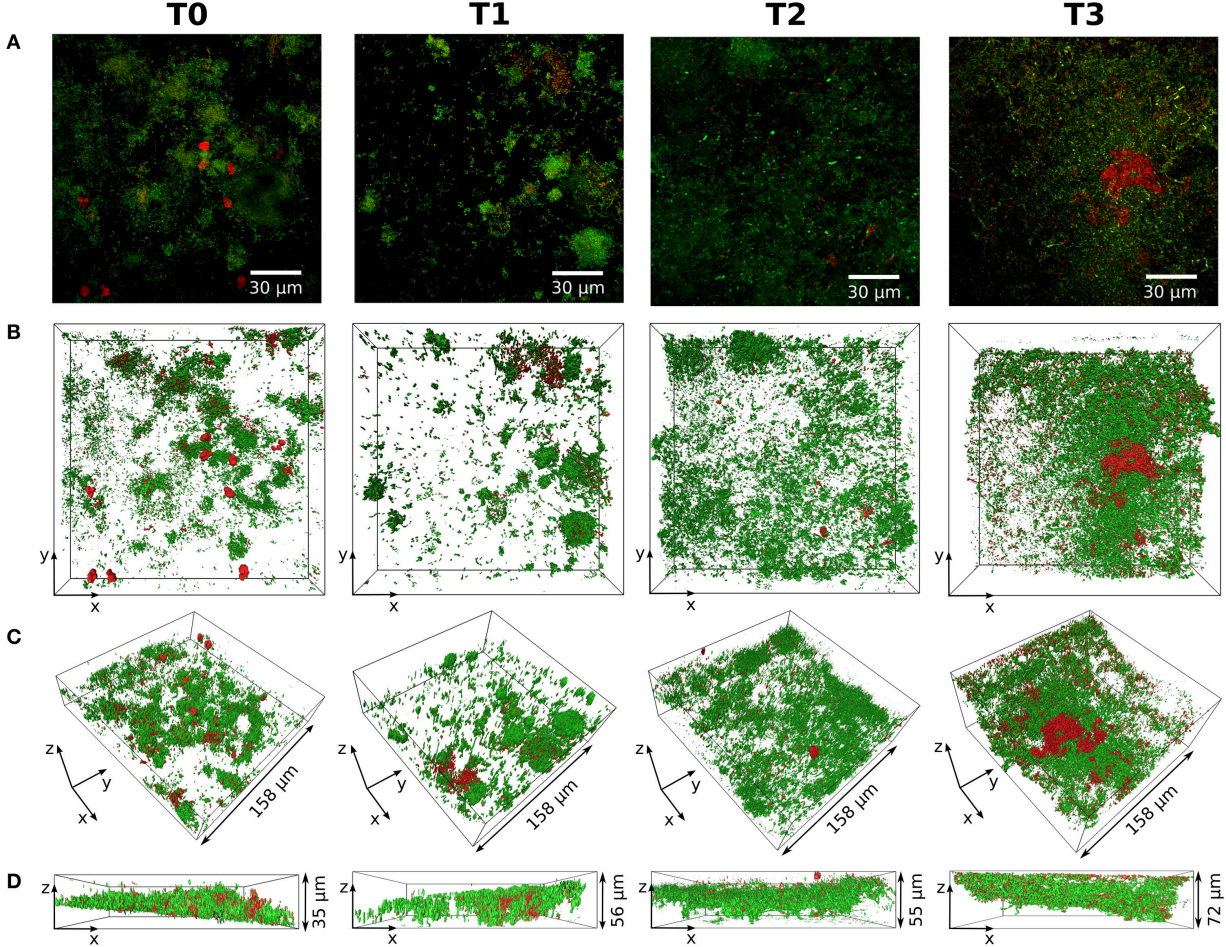
**Structure and composition of the biofilm over time**. Life/dead stained biofilms from one subject (S21) for all points in time (T0–T3) are shown. **(A)** Maximum projections of the respective confocal stacks with living bacteria stained in green and dead bacteria stained in red. The 3D reconstructions are displayed in **(B–D)** with the same coloring as in **(A)**. **(B)** Top view of the 3D reconstruction. **(C,D)**: Side views of the 3D reconstruction.

Figure 3 shows exemplarily one subject's representative live/dead stained 3D reconstruction of confocal stack data over all four sampling times. The figure exemplifies that, on the whole, the relation of living (green) and dead (red) bacteria remained the same at all points in time. An increase in biofilm mass was found at T3. Long chains of coccoid bacteria, probably the *Streptococci* found in the pyrosequencing analysis, on top of large staples of cocci dominated the stacks at T3 as shown in Supplementary Figure 2.

**Figure 4:**
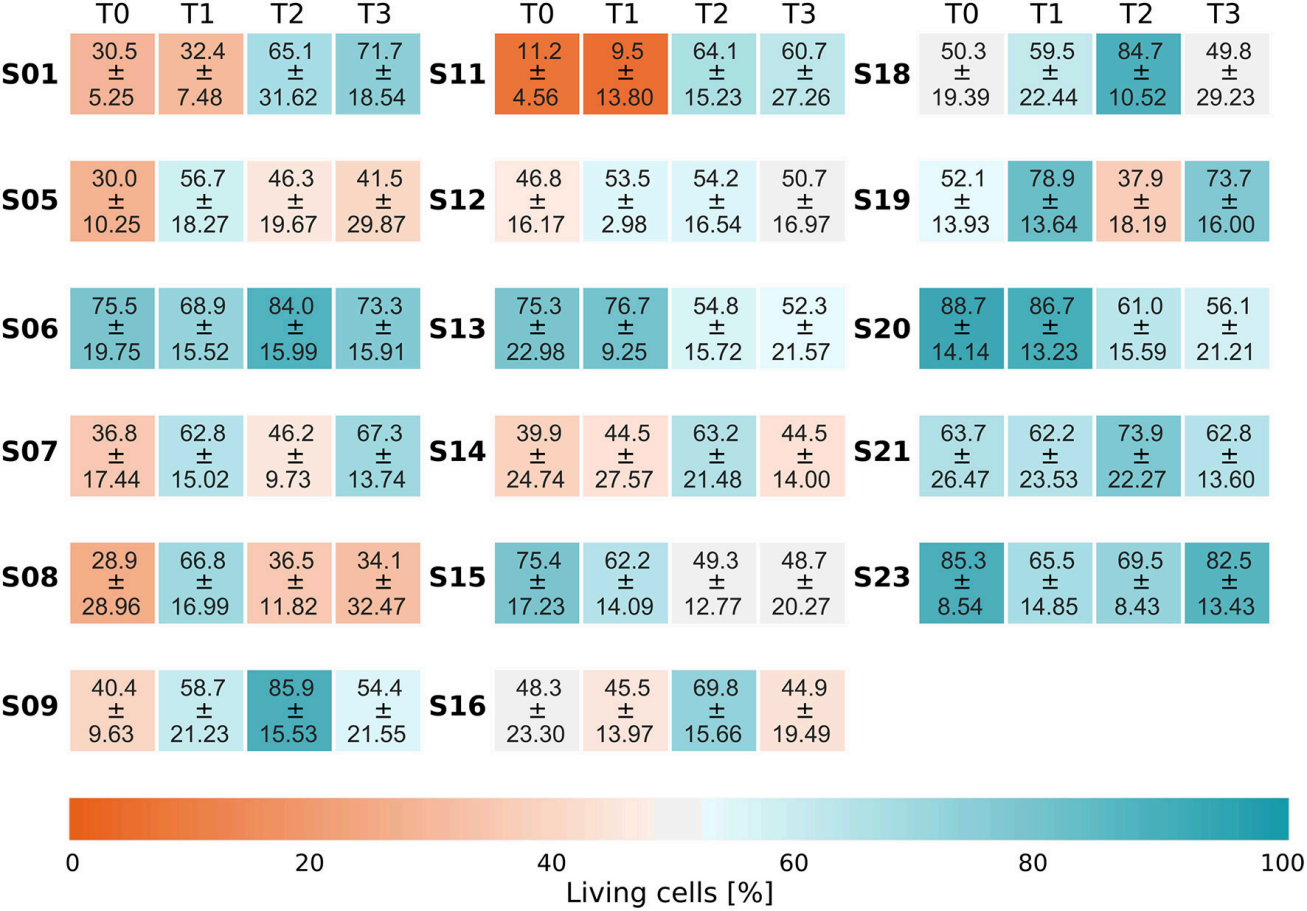
**Life/Dead ratio**. Each time series displays the fraction of living bacteria at each point in time and belongs to one individual subject. Each subject is labeled by a running number (SXX) on the left of the respective time series. The different sampling points in time are displayed by four panels (T0, T1, T2, T3). Values are the average over the fractions of living bacteria ± the sample standard deviation. The respective series plotted individually as a function of time can be found in Supplementary Figure 1.

Survival curves reveal a quite stable growth of microbes inside the biofilm reactor over 48 h (Supplementary Figure 1). Half of the curves show a slight increase in the number of living bacteria during the first 24 h (T2), the other half shows a slight decrease. At T2 (24 h), most curves showed values close to those found at T3. After 48 h, the mean number of living bacteria (blue curve) eventually approximated the initial level measured at T0. The staining for living and dead cells also revealed that a substantial fraction of the natural biofilm contains dead bacteria at T0. Average values and standard deviations of bacterial survival are given in Figure 4.

### Biofilm composition

Compositional shifts during *in vitro* growth at T0 and T3 were revealed with 454 pyrosequencing and are shown in Table 1. Median values of the major phyla found at T0 and T3 were 98.67 and 87.71% for *Firmicutes*, 0.01 and 3.2% for *Bacteroidetes*, 0 and 2.06% for *Proteobacteria*, and finally 0.11 and 0.99% for *Actinobacteria*. *Fusobacteria, Cyanobacteria* and TM7 represented groups with a relative abundance of around 0.1%. SR1, *Spirochaetes*, and *Thermi* were found in very small numbers and in only some samples. The fraction “others” includes all sequences that could not be classified so far.

**Table 1:** **Compositional shifts during *in vitro* growth revealed with 454 pyrosequencing**.

**Taxon: Bacteria**	**Time point 1 [%]**			**Time point 3 [%]**			**Wilcoxon signed-rank test**	**Wilcoxon signed-rank test (Bonferroni corr.)**
**Phylum**	**25th**	**Median**	**75th**	**25th**	**Median**	**75th**	***p*-value^#^**	***p*-value adj.^##^**
Other	0.70	1.07	1.28	1.36	1.69	1.99	0.0007	0.0042
Actinobacteria	0.00	0.11	0.32	0.38	0.99	2.37	0.0001	0.0007
Bacteroidetes	0.00	0.01	0.77	0.70	3.20	8.91	0.0000	0.0002
Firmicutes	97.23	98.67	99.30	82.94	87.71	92.29	0.0001	0.0007
Fusobacteria	0.00	0.00	0.01	0.15	0.26	0.74	0.0000	0.0000
Proteobacteria	0.00	0.00	0.07	0.38	2.06	4.74	0.0019	0.0115

Statistical analysis on phylum level. A p < 0.05 was considered significant after Bonferroni correction.

# *p-value Wilcoxon signed-rank test*.

## *p-value Wilcoxon signed-rank test adjusted according Bonferroni correction*.

**Figure 5:**
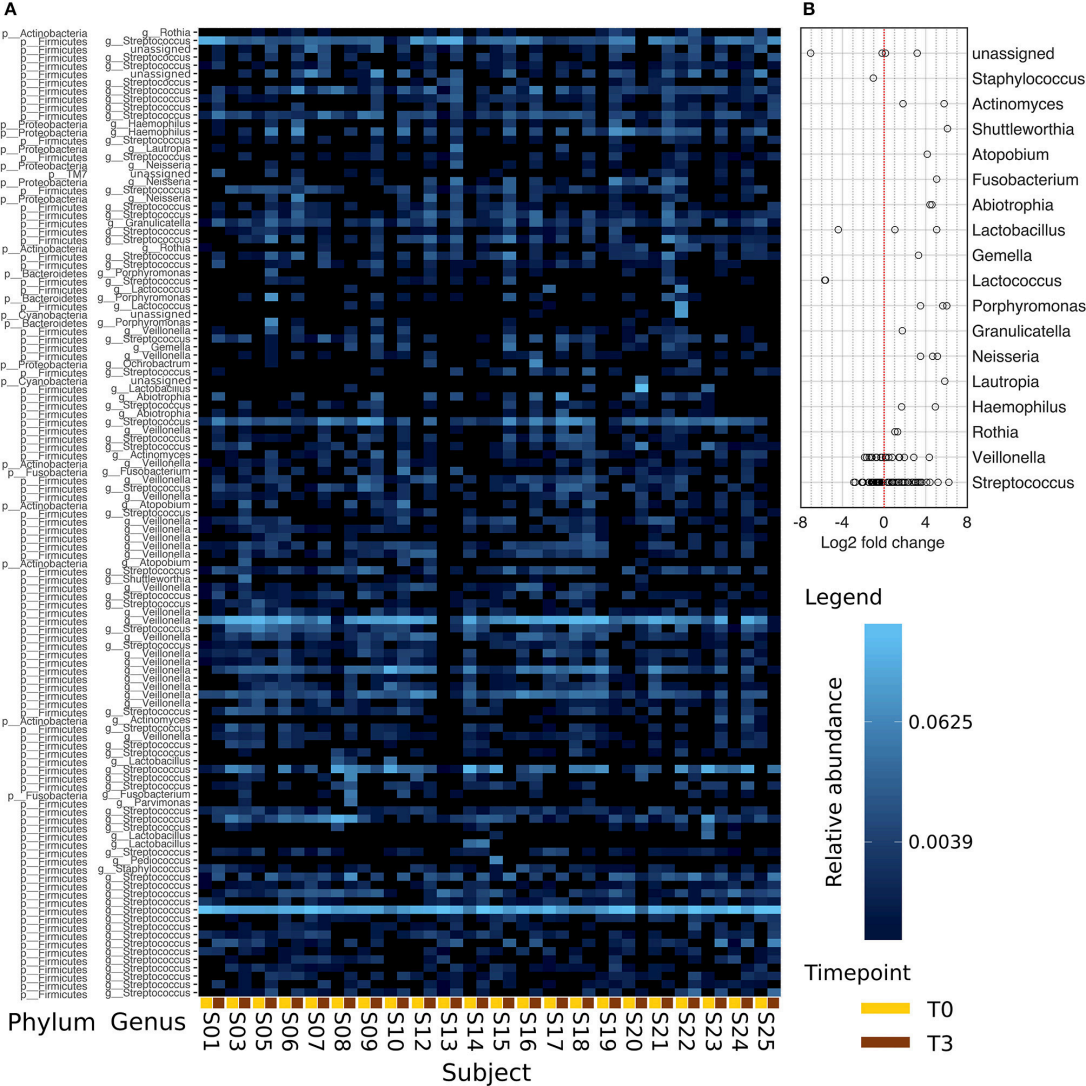
**Comparison of the abundances between T0 and T3**. Data is derived from the 117 most abundant OTUs found in all samples. Panel **(A)** presents this data assigned to the respective genera with a relative abundance >2%. T0 and T3 of each subject are positioned next to each other (line below: T0, yellow; T3, brown). The color scaling is logarithmic. In **(B)**, the log_2_ fold change calculated on the absolute abundances data of **(A)** is plotted. Seven OTUs were excluded as their average abundance in one of the points in time was equal to 0.

Figure 5A shows a heat map of the 117 OTUs common to all samples assigned to genus level with a relative abundance of more than 2%. The heat map is ordered such that T0 and T3 of each subject are plotted next to each other. Samples stay diverse in T3 including anaerobic and aerobic species.

Comparing *in vivo* and *in vitro* growth, the dominating genus found on the enamel-dentin slabs after 48 h of *in vivo* biofilm growth were *Streptococci* (with a mean number of 60.83%) and *Veillonella* (with a 13.38% relative abundance). The dominance of these bacteria remained quite stable even after 48 h of incubation *in vitro* (Table 1). This is also reflected in the log_2_ fold change analysis in Figure 5B. *Lactococci, Lactobacilli*, and *Staphilocci* were found in reduced numbers at T3 while other common oral genera like *Phorphyromonas, Actinomyces, Neisseria* showed a positive log_2_ fold change.

Sample counts analyzed on OTU level with a 97% identity ranged from 1841 to 3863. Assigning this data we found 6 phyla, 9 classes, 12 orders, 15 families, and 17 genera of oral bacteria. A statistical analysis on shifts in bacterial composition over further taxonomic levels is presented in Supplementary Tables 1–4 (excluding values < 0.1% relative abundance). For a better understanding, we added the next higher hierarchical level in parenthesis for unassigned “others” at lower levels. Below we will talk about a significant growth of certain bacteria based on an increase in their relative abundance. All bacterial phyla except *Firmicutes* showed a significant increase over 48 h of *in vitro* incubation. *Firmicutes* decreased significantly. On class level significant changes at T3 could not be found in the two dominating groups, *Bacilli* and *Clostridia*, although their absolute numbers decreased. All other groups with a relative abundance below 3.15% showed over time a relative increase that was statistically significant. *Bacteroidia* and *Gammaproteobacteria* were the two groups with the greatest increase.

No order belonging to the phylum *Firmicutes* showed significant changes at T3, although their numbers decreased. Orders occurring in lower numbers also showed a statistically significant increase in their relative abundance (Supplementary Table 2).

Looking at family levels, *Lachnospiraceae* and *Carnobacteriaceae* in the phylum *Firmicutes* showed a significant increase over time. All the other *Firmicutes* did not change significantly. *Coriobacteriaceae* were the only family in the phylum *Actinobacteria* that increased significantly. *Prevotellaceae* (*Bacteroidales*) and *Pasteurellaceae* (*Gammaproteobacteria*) also grew statistically significantly (Supplementary Table 3).

On genus level only *Actinomyces* and *Rothia* (*Actinobacteria*), *Prevotella* (*Bacteroidetes*), *Granulicatella* (*Firmicutes*), and *Haemophilus* (*Proteobacteria*) showed a significant increase (Supplementary Table 4). All other genera did not show a significant change over the 48 h of incubation in BHI medium.

**Figure 6:**
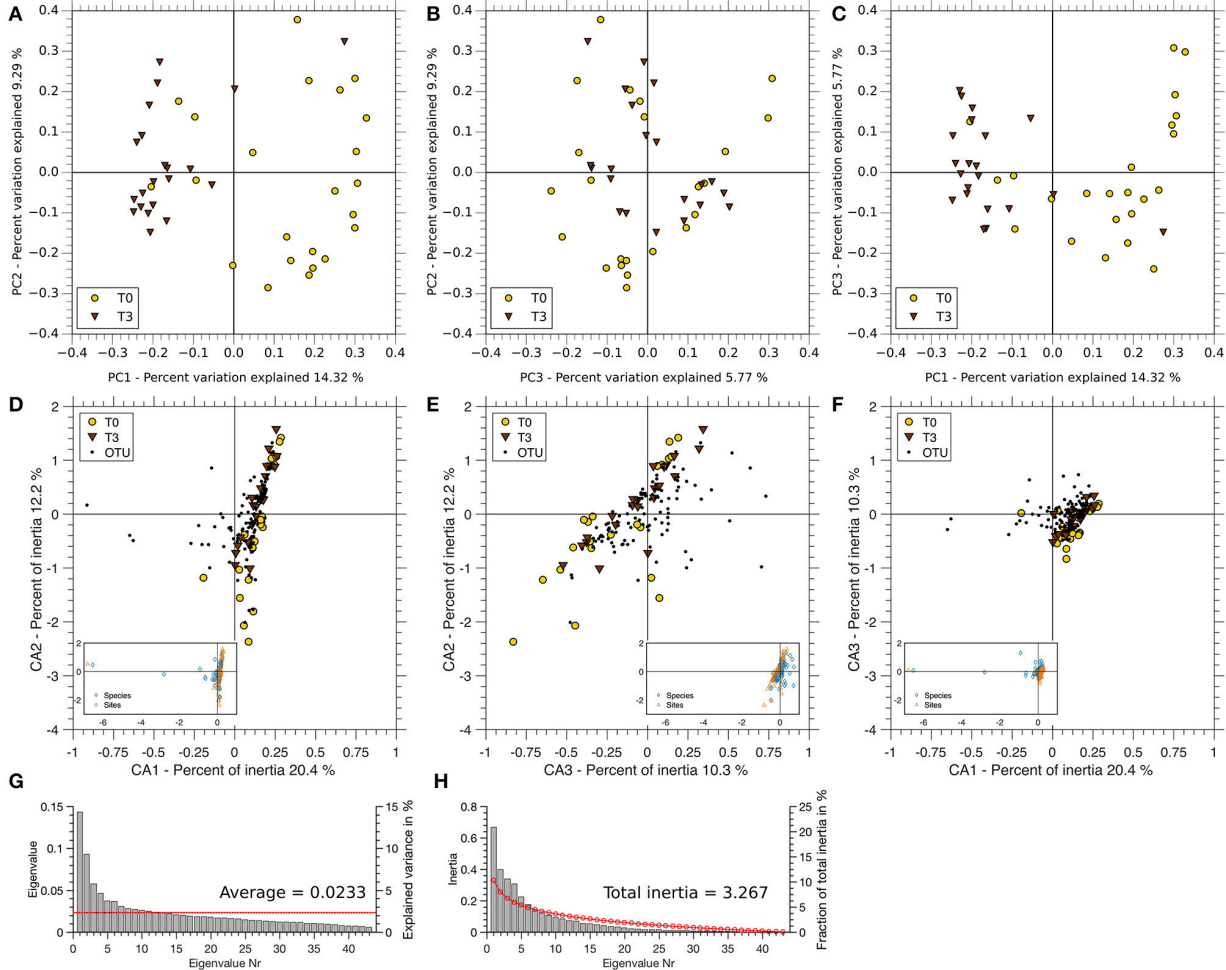
**PCoA and CA comparison of T0 and T3 on genus level. (A–C)** Variance of T0 (yellow) and T3 (brown) pyrosequencing data is shown in the three coordinates (PC1, PC2, PC3) with the highest support (summing up to 29.38%). **(A)** PC1 vs. PC2, **(B)** PC3 vs. PC2, and **(C)** PC1 vs. PC3. **(D,E)** Biplots of OTUs and samples are ordinated by correspondence analysis. **(D)** Axis 1 vs. Axis 2, **(E)** Axis 3 vs. Axis 2, and **(F)** Axis 1 vs. Axis 3. Full plots are shown in the respective small panels of each plot. All plots were scaled with respect to the sites (points in time). **(G)** Scree plot of the Eigenvalues derived by PCoA. The red line indicates the mean over all Eigenvalues. **(H)** Scree plot of the Eigenvalues derived by the CA. The red line shows the broken stick distribution.

A PCoA showed an incomplete clustering of the samples in two levels at T0 vs. T3 (Figures 6A–C). The largest coordinate explains 14.32% of the variation due to time, while the second and the third largest account for 9.29 and 5.77%, respectively. Approximately 70% of the variation is due to other factors. A clustering of T0 and T3 can be seen in Figures 6A,C. Correspondence analysis showed an even distribution of subjects at T0 and T3 (Figures 6D–F). No clustering of one of the points in time was found. The first three axes of the CA explain 20.4, 12.2, and 10.3% of the total inertia of the respective data. There is an even distribution of T0 and T3 samples in all dimensions shown. The respective OTUs appear near the samples.

The distribution of Eigenvalues explaining the variance and the fraction of total inertia are shown in Figure 6G (PCoA) and Figure 6H (CA), respectively.

### Fluorescence *In situ* hybridization

**Figure 7:**
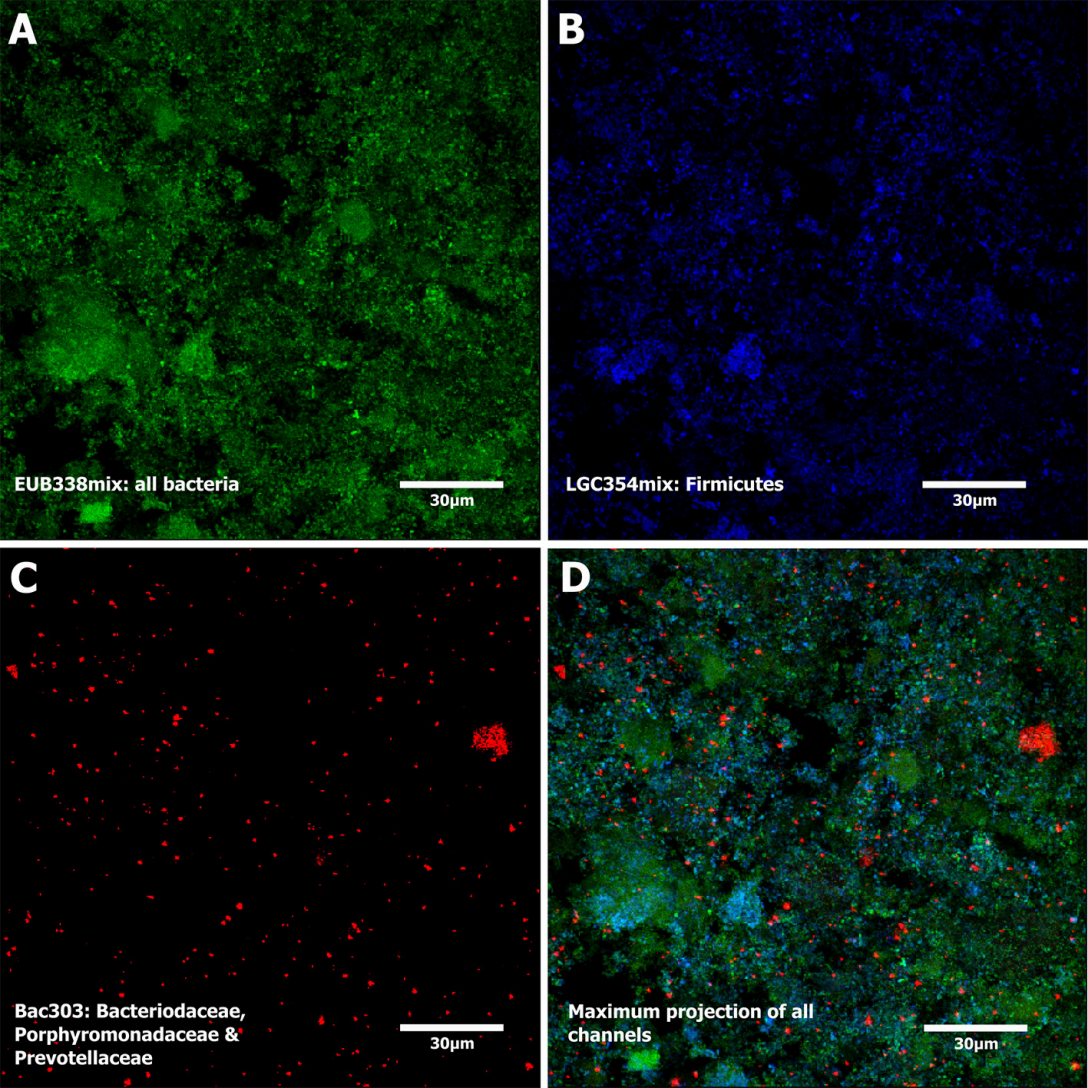
**Fluorescence *in situ* hybridization**. The recorded color channels are shown in **(A–C)** and are maximum projections of a confocal stack. **(A)** Biofilm stained with EUB338mix (green, all bacteria), **(B)** LGC354mix (blue, Firmicutes), and **(C)** Bac303 (red, Bacteroidaceae, some Porphyromonadaceae, and Prevotellacea). Panel **(D)** shows a maximum projection of all 3 channels. The presented biofilm was sampled at T3.

FISH analysis showed clear signals of all bacteria stained in green (EUB338mix with Cy3) and *Firmicutes* (EUB338mix with Cy3 and LGC354mix with FITC) in light blue (Figure 7). The majority of bacteria was stained in light blue confirming the pyrosequencing results where *Bacilli* and *Clostridia*, both *Firmicutes*, represented the largest groups. Signals could also be detected from probe Bac303 representing *Bacteriodaceae*, and some *Porphyromonadaceae* and *Prevotellaceae* (red signal).

## Discussion

Modeling native oral biofilm growth is tricky, as many different taxa play a role in co-aggregation and bacterial succession. In order to extend knowledge on initial biofilm colonization, many studies have stained native oral biofilm, e.g., with FISH (Thurnheer et al., 2004; Hannig et al., 2007; Jung et al., 2010; Zijnge et al., 2010). Some of these studies used biofilm sampled directly from the oral cavity, some of them used carrier materials on which biofilm was grown. Hydroxyapatite discs were often chosen to simulate the tooth surface and to study primary colonization. Different attempts were made using *in vitro* and *in vivo* assays (Walker and Sedlacek, 2007; Guggenheim et al., 2009; Ledder et al., 2009; Rudney, 2012). Hannig et al., for example, fixed individual splints in the upper jaw with bovine enamel discs as biofilm carriers (Hannig et al., 2007). They found an initial colonization of Streptococci as early as after 3 min. These first studies were based on the analysis of the primary colonization and co-aggregation of bacteria on hydroxyapatite or bovine enamel. They explored biofilm formation and composition directly after removing the sample from the oral cavity. We have gone a step further. Our aim was to improve previous systems by, firstly, using real human enamel-dentin slabs as biofilm carriers, secondly, transferring the biofilm to the laboratory without any disturbance, and, thirdly, keeping the biofilm alive under *in vitro* conditions. Based on the dental splints used in Jung et al. and Al-Ahmad et al., we designed a dental splint carrying the human enamel-dentin slabs of a standardized size and grid (Al-Ahmad et al., 2007; Jung et al., 2010). Growing the biofilm directly in the human mouth on human enamel-dentin slabs leads to the formation of a native biofilm which is normally found in the supragingival area after pellicle formation (Nobbs et al., 2011; Teles et al., 2012; Jakubovics, 2015). The dental splint developed in our study enables us to insert up to six enamel-dentin slabs measuring 4 × 6 mm. As the slabs are positioned adjacent to the supragingival area, the biofilm finds similar conditions to those encountered directly on the individual's tooth. Although the slabs are sheltered from strong shear forces, saliva can bathe them and nourish the biofilm. Waste products can be washed away, as they would be under natural conditions. In our study, dental splints were carried intraorally for continuous 48 h. This way we could enrich a native primary biofilm directly in the oral cavity under native conditions (“from mouth”) prior to transfer to the laboratory (“to model”). The easy accessibility of the enamel-dentin slabs in the dental splint allows for a quick transfer to *in vitro* systems without any disturbance of the biofilm caused by temperature shifts, excess oxygen or other factors.

For our experiments we used BHI medium as an alternative to real saliva, as it is similar to sulcus fluid (Standar et al., 2010). The quality of initially used sterilized human saliva treated with 2.5 mM Dithiothreitol (Foster et al., 2004) was uncontrollable regarding workflow standardization and resulted in almost complete loss of viability in most attempts. In contrast, BHI works for both, anaerobes and aerobes, and thus was deemed an appropriate surrogate for our experiments, as we aimed to keep the model standardized.

Treating the biofilm with the LIVE/DEAD® BacLight™ Bacterial Viability Kit directly on the enamel-dentin slabs and using water immersible lenses for microscopy avoided damage to the biofilm structure. This was useful for detecting larger structures in the transferred biofilm. We observed (>100 μm) long chains of cocci at T3, similar to those reported in direct observations of oral biofilms. Living and dead cells frequently coexist. Looking at the survival of bacteria, the curves showed variable courses but, all in all, the proportion of living and dead bacteria after 48 h of incubation approximated the level observed at T0. In the biofilm reactor the bacteria found stable temperature conditions and BHI as a very rich food source, leading to perturbation at T1. Already after 24 h, live/dead proportions appeared to be almost at the level seen at T0 again. It seems as if the perturbation in biofilm growth at T1 originated from the transfer to the biofilm reactor. Finally, at 48 h, the initial proportions of living and dead bacteria were reached. We therefore conclude that the biofilm in our system stayed vital for at least 48 h *in vitro*. Netuschil et al. (2014) reported that several groups had analyzed the staining behavior of the LIVE/DEAD® BacLight™ Bacterial Viability Kit and other live/dead staining kits. SYTO 9 and PI work best with a previously tested mixture for each individual bacterium. These tests are not feasible when working with natural biofilms comprised of several hundred species, because sometimes both stains penetrate the same cell. Therefore, a compromise has to be made for such samples. For proper evaluation of these ambiguously stained cells, however, the obtained images have to be analyzed carefully. In our study, we excluded orange (red + green) signals from further analyses. We are aware that this might have led to an underestimation of viable cells. As there is no software available that consistently excludes the misleading signals, a specific MATLAB script was designed for this purpose.

In our analysis of the biofilm composition, the phylum *Firmicutes*—and here *Streptococci* (facultative anaerobes) and *Veillonella* (anaerobes)—appear to be the primary colonizers forming a “base” on which other bacteria can dock (Rickard et al., 2003; Zijnge et al., 2010). *Streptococci* spp. and *Veillonella* spp. have been reported to show a strong co-occurrence and co-aggregation in native oral biofilm and to interact in *in vitro* tests (Egland et al., 2004; Palmer et al., 2006; Chalmers et al., 2008; Santigli et al., 2016). They also showed a similar behavior over the 48 h of incubation as demonstrated in the log_2_ fold change analysis. This leads us to the assumption that those two genera further interact in our *in vivo* system. We also found that *Streptococci* made up the biggest bacterial group with around 60% of the population. These did not change significantly in number after 48 h of *in vitro* incubation. Interestingly, other genera like *Actinomyces* (mainly anaerobic growth), *Prevotella* (obligate anaerobes) and *Rothia* (facultative anaerobes) increased significantly. Kolenbrander et al. (2006) showed co-aggregation of Actinomyces naeslundii *T14V* with *Streptococcus, Prevotella* (obligate anaerobes), and *Capnocytophaga* strains. These genera play an important role in the “pre-organization” phase of the biofilm which is the period in biofilm development lasting between 18 h and up to 4 days (Jakubovics, 2015). Together with *Streptococci* and *Veillonella* they also tended to remain the predominant microorganisms although their relative abundance stagnated (Diaz et al., 2006). This increase after several days has been previously shown *in vivo* by Takeshita et al. (2015). The growing numbers seen in the other genera, i.e., facultative and obligate anaerobes, prove that our *in vitro* model using the BHI medium works without an anaerobic chamber. The ability to keep these genera alive over several generations is a good foundation for further assays. This is also supported by a heat map analysis on OTU level. The α-diversity calculated with PD-whole tree is higher across all samples at T3. Sterility was proven for the biofilm reactor system, so we can argue that the reason for higher values at T3 is a relative abundance at T0 which was too low to be detected by 454 pyrosequencing. As the biofilm sampling is discontinuous due to two different slabs used for T0 and T3, bacteria found at T3 can also derive from this.

PCoA explaining around 30% of the variance due to time shows a clustering of the points in time in two dimensions, no clustering can be found in the third dimension. To be able to better interpret this environmental data, correspondence analysis was used to model the change between T0 and T3 and the OTU distributions based on the same data as PCoA. The CA shows a clear proximity of the samples at T0 and T3 with around 40% of total inertia. OTUs appear in high abundance at both points in time reflected through the data points shown in strong vicinity to the sample points. Correspondence analysis thus supports our hypothesis that there is no difference between T0 and T3.

To get more information on cell viability and to confirm the biofilm composition found by pyrosequencing, we also performed the FISH analysis. FISH probes that only bind to viable cells prove that our biofilm is not only vital, but still able to live. Based on the strong signals gained, we conclude that the biofilm is also vital. LGCmix, staining *Firmicutes*, represented the main group also in FISH analysis. This is consistent with our pyrosequencing data showing *Firmicutes* as the largest group. Furthermore, signals were recorded from Bac303 staining most *Bacteroidaceae* and *Prevotellaceae*, and some *Porphyromonadaceae*. This result goes along with previous findings that detected these groups in healthy adults (Aas et al., 2005).

Our “mouth to model” system allows for native oral biofilm growth *in vivo*, a simple transfer of this biofilm to laboratory setups and further growth *in vitro* in biofilm reactors. Our setup can be easily reconstructed and settings used in miscellaneous studies. With this setup the biofilm stays alive and diverse over 48 h of *in vitro* incubation. This is an important outcome making our study a sound basis for a new biofilm model to be used in (phyto-) pharmacological assays or dental materials research. Further investigations and validation of the appropriate conditions for *in vitro* cultivation of native oral biofilms could facilitate the study of all biofilm-induced diseases.

## Author contributions

BK: work conception and design; acquisition, analysis and interpretation of data; drafting and critical review of the manuscript; final approval of the work for publication. ES: study design; analysis and interpretation of data; draft and critical review of the manuscript; final approval of the work for publication. CW: analysis and interpretation of data; critical review of the manuscript; final approval of the work for publication. ST: analysis and interpretation of data; critical review of the manuscript; final approval of the work for publication. GW: analysis and interpretation of data; critical review of the manuscript; final approval of the work for publication. MG: analysis and interpretation of data; critical review of the manuscript; final approval of the work for publication.

## Funding

We gratefully acknowledge the support by “Land Steiermark” (Human Technology Interface grant: OraSim).

### Conflict of interest statement

The authors declare that the research was conducted in the absence of any commercial or financial relationships that could be construed as a potential conflict of interest.

## Supplementary material

Supplementary Table 1 **Statistical analysis on class level**. A p < 0.05 was considered significant after Bonferroni correction.

Supplementary Table 2 **Statistical analysis on order level**. A p < 0.05 was considered significant after Bonferroni correction.

Supplementary Table 3 **Statistical analysis on family level**. A p < 0.05 was considered significant after Bonferroni correction.

Supplementary Table 4 **Statistical analysis on genus level**. A p < 0.05 was considered significant after Bonferroni correction.

Supplementary Video 1 **Analysis of the recorded biofilm and exclusion of suspected yeast cells**. The movie shows an entire confocal stack of a life/dead stained biofilm and its corresponding analysis. **(A)** Living bacteria stained in green. **(B)** Dead bacteria and suspected yeast cells stained in red. The boundary of the detected foreground of **(A,B)** is displayed as a green line **(C)** and an orange line **(D)**, respectively. Additionally, the purple line shows the boundary of the manually selected yeast. These regions were excluded from further analysis.

Supplementary Figure 1 **Evolution of the Life/Dead Ratio over time**. Each individual plot displays the evolution of the fractions of living and dead bacteria over time. Each plot belongs to one subject and the running number (SXX) is given on the top left corner. Within an individual plot, each data point represents the mean over all fractions of living (top plot) and dead bacteria (bottom plot) at the particular point in time. The colored region gives the sample standard deviation.

Supplementary Figure 2 **Structure of the biofilm**. Exemplary images of life/dead stained biofilms with the different observed structures. Each image is the maximum projection of the respective recorded confocal stack. Often observed structures are cocci filaments **(A,C)** and cocci staples **(A,B)**.
