# Supplementary figures and images for: From Mouth to Model: Combining in vivo and in vitro Oral Biofilm Growth
Source: Front Microbiol. 2016 Sep 21;7:1448. doi: 10.3389/fmicb.2016.01448 (PMC5030783; doi:10.3389/fmicb.2016.01448)

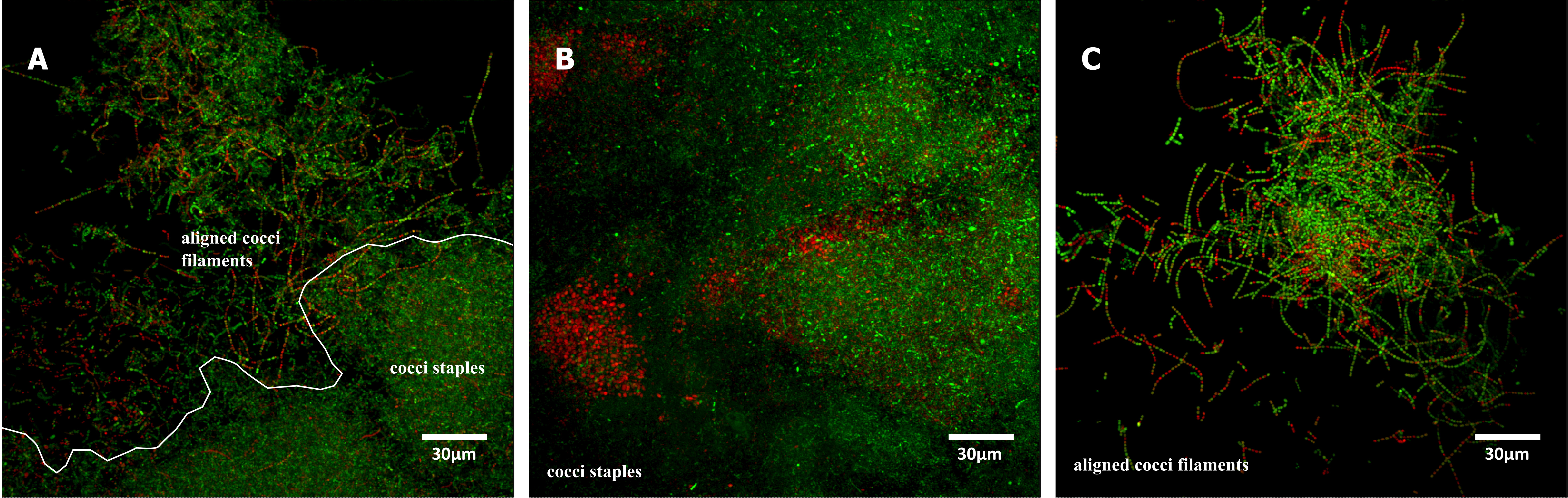

Supplement: Supplementary Figure 2 — Structure of the biofilm. Exemplary images of life/dead stained biofilms with the different observed structures. Each image is the maximum projection of the respective recorded confocal stack. Often observed structures are cocci filaments (A,C) and cocci staples (A,B). [file Image2.JPEG]
